# Predictive Biomarkers for Antipsychotic Treatment Response in Early Phase of Schizophrenia: Multi-Omic Measures Linking Subcortical Covariant Network, Transcriptomic Signatures, and Peripheral Epigenetics

**DOI:** 10.3389/fnins.2022.853186

**Published:** 2022-05-09

**Authors:** Xiaofen Zong, Changchun He, Xinyue Huang, Jinming Xiao, Lei Li, Meiling Li, Tao Yao, Maolin Hu, Zhongchun Liu, Xujun Duan, Junjie Zheng

**Affiliations:** ^1^Department of Psychiatry, Renmin Hospital of Wuhan University, Wuhan, China; ^2^The High-Field Magnetic Resonance Brain Imaging Key Laboratory of Sichuan Province, University of Electronic Science and Technology of China, Chengdu, China; ^3^Department of Radiology, The Athinoula A. Martinos Center for Biomedical Imaging, Harvard Medical School, Massachusetts General Hospital, Boston, MA, United States; ^4^The Early Intervention Unit, Department of Psychiatry, Affiliated Nanjing Brain Hospital, Nanjing Medical University, Nanjing, China; ^5^The Functional Brain Imaging Institute, Nanjing Medical University, Nanjing, China

**Keywords:** schizophrenia, antipsychotic, subcortical covariant network, DNA methylation, MRI, Allen Human Brain Atlas

## Abstract

**Background:**

Volumetric alterations of subcortical structures as predictors of antipsychotic treatment response have been previously corroborated, but less is known about whether their morphological covariance relates to treatment outcome and is driven by gene expression and epigenetic modifications.

**Methods:**

Subcortical volumetric covariance was analyzed by using baseline T1-weighted magnetic resonance imaging (MRI) in 38 healthy controls and 38 drug-naïve first-episode schizophrenia patients. Patients were treated with 8-week risperidone monotherapy and divided into responder and non-responder groups according to the Remission in Schizophrenia Working Group (RSWG). We utilized partial least squares (PLS) regression to examine the spatial associations between gene expression of subcortical structures from a publicly available transcriptomic dataset and between-group variances of structural covariance. The peripheral DNA methylation (DNAm) status of a gene of interest (GOI), overlapping between genes detected in the PLS and 108 schizophrenia candidate gene loci previously reported, was examined in parallel with MRI scanning.

**Results:**

In the psychotic symptom dimension, non-responders had a higher baseline structural covariance in the putamen–hippocampus–pallidum–accumbens pathway compared with responders. For disorganized symptoms, significant differences in baseline structural covariant connections were found in the putamen–hippocampus–pallidum–thalamus circuit between the two subgroups. The imaging variances related to psychotic symptom response were spatially related to the expression of genes enriched in neurobiological processes and dopaminergic pathways. The DNAm of GOI demonstrated significant associations with patients’ improvement of psychotic symptoms.

**Conclusion:**

Baseline subcortical structural covariance and peripheral DNAm may relate to antipsychotic treatment response. Phenotypic variations in subcortical connectome related to psychotic symptom response may be transcriptomically and epigenetically underlaid. This study defines a roadmap for future studies investigating multimodal imaging epigenetic biomarkers for treatment response in schizophrenia.

## Introduction

Early treatment response is proposed to be one of the robust predictors of schizophrenia patients’ functional outcomes (Lambert et al., 2008), but it is variable and cannot be precisely predicted by the treating physician. Hence, it is critical to ascertain biomarkers with a predictive potential by investigating their relation to prospective treatment response in the early phase of schizophrenia.

Subcortical structures, including the basal ganglia and some areas of the limbic system, have attracted interest in schizophrenia research (Okada et al., 2016; Van Erp et al., 2016). They contain abundant dopaminergic neurons, the disruption of which is thought to be related to schizophrenia pathology and the formation of psychotic symptoms such as hallucinations and delusions (Howes and Kapur, 2009). Moreover, the remarkable 108 candidate gene loci of schizophrenia identified previously (Schizophrenia Working Group of the Psychiatric Genomics, 2014) also include many genes relevant to dopamine pathways. These studies link both subcortical structure phenotypes and schizophrenia pathology to the dopamine signaling pathways. Moreover, a recent large-scale imaging-genetic study detected the genetic overlap between schizophrenia risk and volumes of subcortical structures including the hippocampus and putamen using genome-wide association study (GWAS) tools (Smeland et al., 2018). The evidence above implies common genetic associations emerging for both subcortical structure phenotypes and schizophrenia pathology. In addition, subcortical structures are the main targets of antipsychotic drugs, and their neuroimaging measurement alterations have also been suggested to be in relation to a lack of response to neuroleptic treatment (Sarpal et al., 2016; Li et al., 2020).

In the domain of neuroimaging, functional connectivity measures of the subcortical areas have been suggested to be an excellent marker for predicting antipsychotic treatment outcomes (Sarpal et al., 2016; Li et al., 2020). The volume of the subcortical structures also shows predictive potential for neuroleptic treatment response (Buchsbaum et al., 2003; Strungas et al., 2003). However, less is known about the predictive role of their structural connectivity patterns. The principal imaging approach available for this purpose is the structural covariance analysis of T1-weighted magnetic resonance imaging (MRI). The “structural covariance,” i.e., morphological correlation, is an organizational pattern in the brain measured across a population and assesses the statistical associations of pairs of brain regions in their anatomical properties such as volume (Alexander-Bloch et al., 2013a,b). Researchers have demonstrated the abnormalities of structural covariance in Parkinson’s disease (Chou et al., 2015; De Schipper et al., 2017; Xu et al., 2017), a neurodegenerative disease caused by dopamine deficiency in the basal ganglia, suggesting the potential associations between structural covariance abnormalities and dopamine signals. Moreover, a positron emission tomography (PET) study revealed the relationship between the baseline dopamine synthesis capacity of subcortical regions and improvements in positive and negative symptoms after treatment (Jauhar et al., 2019). These previous studies led us to speculate that subcortical structural covariance before starting treatment may underlay future variation in response to antipsychotic treatment. Yet, few studies have directly investigated how baseline subcortical morphological covariance relates to treatment outcome in the early phase of schizophrenia.

Another unsolved issue is that less is known about whether the variance of subcortical morphological connectivity is driven by gene expression patterns and epigenetic mechanisms, which are the plausible molecular basis of phenotypic heterogeneity across individuals with schizophrenia. Epigenetic modifications are proposed to mediate between environmental insults and gene expression and alter and stably maintain the expression of genes (Magwai et al., 2021). DNA methylation (DNAm) is the most widely explored epigenetic mechanism and has been suggested to be associated with an antipsychotic drug action mechanism (Ovenden et al., 2018). Although DNAm is to a degree tissue-specific, 10.9% of DNAm sites were moderately robustly associated (*r* > 0.5) between brain and blood (Braun et al., 2019), implying that DNAm status of peripheral blood cells may act as a surrogate for that of central tissues. Noteworthily, structural covariance is thought to be specifically related to co-expression of a set of genes relevant to neurobiological processes (Romero-Garcia et al., 2018), which suggests the heritability of inter-regional structural covariation. Recent brain expression atlases bridge the gap between epigenetic modifications and brain connectome phenotypes. The Allen Human Brain Atlas (AHBA), a publicly available transcriptomic dataset (Hawrylycz et al., 2012), has been utilized to identify transcriptomic signatures associated with brain network connectivity of individuals with mental disorders, such as schizophrenia and major depressive disorder (MDD) (Morgan et al., 2019; Li et al., 2021), which uncovers the molecular foundation of regional brain vulnerability to major mental disorders. Combining brain network phenotypes, brain gene expression, and DNAm is imperative in the developing of baseline multi-omic biomarkers associated with treatment outcome, as it can integrate multiple omics data to comprehensively understand efficacy heterogeneity across individuals, although few such studies have been conducted.

This current study, therefore, integrates multi-omic measures including subcortical covariance network, AHBA transcriptome data, and peripheral DNAm to comprehensively understand efficacy heterogeneity across individuals. Patients were treated with 8-week risperidone monotherapy to control for the effects of confounders related to multidrug therapy on treatment response. They were divided into responders and non-responders according to the Remission in Schizophrenia Working Group (RSWG) (Andreasen et al., 2005). We applied a seed-based multivariate technique to identify the patterns of subcortical structural covariance in responders and non-responders. Based on previous evidence (Buchsbaum et al., 2003; Strungas et al., 2003; Xu et al., 2017; Ovenden et al., 2018; Romero-Garcia et al., 2018; Yee et al., 2018), it was hypothesized that: (1) patients’ baseline subcortical structural covariance would be associated with patients’ treatment response; (2) variances of baseline subcortical structural covariance between responders and non-responders would be spatially correlated with brain gene expression acquired from the AHBA, and these genes would be enriched in dopaminergic pathways and neurobiological processes; and (3) the DNAm levels of gene of interest (GOI) would also relate to patients’ treatment response. The GOI was defined as the overlapping genes of AHBA genes spatially correlated with baseline brain measures and the 108 schizophrenia candidate loci (Schizophrenia Working Group of the Psychiatric Genomics, 2014), as they were associated with both the schizophrenia pathology and variations in subcortical structural covariance related to treatment response.

## Materials and Methods

### Participants

We recruited 38 treatment-naive first-episode schizophrenia patients and 38 gender-, age-, and education-matched healthy controls from October 2012 to January 2014 in Henan Mental Hospital, Xinxiang, China. This dataset was previously used by our group (Hu et al., 2016a,b; Zong et al., 2019). Patients were diagnosed by experienced psychiatrists by using the Structured Clinical Interview for the Diagnostic and Statistical Manual of Mental Disorders, 4th Edition, Text Revision (DSM-IV-TR). All patients’ disease duration was not more than 12 months. Healthy volunteers without a history of neurological and psychiatric disorders were screened by using the Structured Clinical Interview for DSM Disorders (SCID)-non-patient edition. All procedures in this study were approved by the ethics committee (No. S088, 2012) of the Second Xiangya Hospital. Details about patients’ selection, preparation and testing, antipsychotic therapy, clinical behaviors, and treatment response assessment were shown in Supplementary Materials and Methods.

### Three-Step Multi-Omic Analysis

**FIGURE 1:**
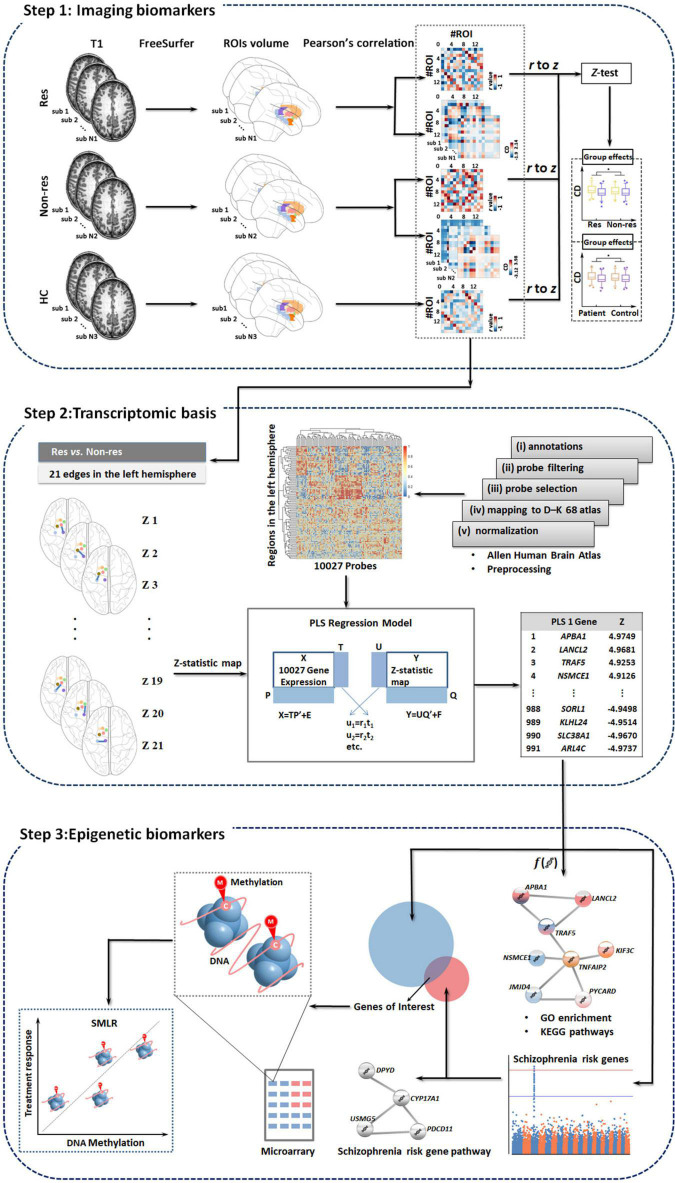
Study overview. We develop baseline biomarkers associated with treatment outcomes using multi-omic measures linking subcortical covariant network, transcriptomic signatures, and peripheral epigenetic modifications based on three steps. Res, responders; Non-res, non-responders; HC, healthy controls; PLS, partial least squares; GO, gene ontology; KEGG, Kyoto encyclopedia of genes and genomes; SMLR, stepwise multiple linear regression.

We constructed baseline biomarkers associated with treatment outcome using multi-omic measures linking subcortical covariant network, transcriptomic signatures, and peripheral epigenetic modifications based on the following three steps (Figure 1) in psychotic, disorganized, and total symptom dimensions except for negative symptoms (as patients did not show significant improvement in negative symptoms after treatment, data shown in Table 1).

**TABLE 1:** Demographics and clinical symptoms for healthy volunteers and patients both at baseline and follow-up.

Variables	Patients at baseline (*n* = 38)	Patients at follow-up (*n* = 38)	Healthy controls (*n* = 38)	*t*/χ^2^^a^	*p*
Age (years), Mean ± *SD*	25 ± 4.95	NA	24.76 ± 4.56	*t*_(74)_ = 0.22	0.83
Education (years), Mean ± *SD*	10.39 ± 2.86	10.39 ± 2.86	11.05 ± 2.91	*t*_(74)_ = −0.99	0.32
Duration of psychosis (months), Mean ± *SD*	8.23 ± 2.60	NA	NA	NA	NA
Handedness (R/L)	38/0	38/0	38/0	NA	NA
Gender (male/female)	25/13	25/13	25/13	χ^2^_(1)_ = 0	1
PANSS-T	92.89 ± 10.96	66.32 ± 9.91	NA	*t*_(37)_ = 19.15	*p* < 0.001
PANSS-P	25.97 ± 3.61	15.39 ± 2.98	NA	*t*_(37)_ = 19.15	*p* < 0.001
PANSS-N	18.32 ± 5.09	16.76 ± 4.45	NA	*t*_(37)_ = 1.94	*p* = 0.06
PANSS-G	48.61 ± 6.52	34.16 ± 4.84	NA	*t*_(37)_ = 13.47	*p* < 0.001

*L, Left; R, right; PANSS, Positive and Negative Syndrome Scale; PANSS-T, PANSS total symptoms; PANSS-P, PANSS positive symptoms; PANSS-N, PANSS negative symptoms; PANSS-G, PANSS general psychopathology symptoms; SD, standard deviation; NA, not applicable.*

*^a^t_(df)_, Between-group t-statistic and degrees of freedom. χ^2^_(df)_, Between-group chi-square statistic and degrees of freedom.*

#### Step 1: Subcortical Covariant Network Biomarkers of Treatment Response

##### T1 Imaging Acquisition

Data were scanned on a 3.0-T Siemens MRI scanner (Verio) at the Magnetic Imaging Centre of the Henan Mental Hospital. Both patients and controls underwent a single baseline neuroimaging assessment (for more details on acquisition parameters, see Supplementary Information).

##### Image Analysis

Subcortical volume estimation was automatically performed with the publicly available FreeSurfer software package by using the program segmentation procedure (v5.3.0)^1^ (for more details on image processing, see Supplementary Information).

##### Extraction of Seed Volumes

Based on our hypothesis, our analysis concentrated on the following 14 subcortical regions of interest (ROIs): the bilateral thalamus, amygdala, hippocampus, caudate nucleus, putamen, pallidum, and nucleus accumbens. Each of the 14 regions was defined by the automated FreeSurfer segmentation procedure and extracted from the bilateral hemispheres. To adjust for the different brain sizes of each subject, we also extracted the estimated total intracranial volume (TIV).

##### Structural Covariance Analysis

Structural covariance was defined as Pearson’s correlation of brain volumes between anatomical ROIs across subjects, simultaneously controlling for the nuisance effects of age, gender, and TIV through partial correlation analysis. To examine how structural covariance related to the response for psychotic, disorganized, and total symptom dimensions, we constructed the baseline structural covariance matrix in responders and non-responders, respectively, for each symptom group. The value of each structural covariance connectivity (*r*_*i*_, i.e., Pearson’s correlation coefficient) was then converted to *Z*_*i*_ by using Fisher’s *r*-to-*z* transformation, which was performed as follows:


$$Z_{i}=\frac{1}{2}\,\log_{e}\frac{1+r_{i}}{1-r_{i}}$$

We applied the *Z*-test in the baseline subcortical structural covariance matrices to determine the group differences in each symptom dimension. The value of *Z*_*i*_ approximately follows the normal distribution with variance equal to 1/(*N* − 3), where *N* is the sample size. Thus, the *Z*-test comparing between-group differences of subcortical structural covariance connectivity was performed as follows:


$$Z=\frac{Z_{A}-Z_{B}}{\sqrt{\frac{1}{N-3}+\frac{1}{M-3}}}$$

where *N* and *M* are the sample sizes of the groups A and B. Then, the *p*-value was calculated through the cumulative distribution function of the *Z*-test. Multiple comparisons were corrected by the false discovery rate (FDR), and the level of statistical significance was set at *p* < 0.05. All the calculation was programmed in-house.

#### Step 2: Transcriptomic Basis of Imaging Biomarkers

##### Preprocessing of Allen Human Brain Atlas Data

Transcriptional profiles, including 20,737 gene expression data represented by 58,692 probes, were obtained from the AHBA^2^ (Hawrylycz et al., 2012). The expression data were preprocessed according to the previously reported five major steps (Arnatkeviciute et al., 2019). Further details concerning preprocessing are provided in Supplementary Material. We only included tissue samples in the left hemisphere, as all the six donors have tissue samples in the left hemisphere, whereas only two donors had samples in the right hemisphere (Arnatkeviciute et al., 2019). After the above five steps of preprocessing, there were 10,027 probes for each sample.

To extract post-mortem brain tissue samples spatially located in the subcortical structures, we only selected samples (1) that had corresponding structure annotation concerning the subcortical structures provided by the AHBA ontology and (2) whose Montreal Neurological Institute (MNI) coordinates could be precisely mapped to the subcortical regions. The mean expression value of all brain tissue samples in a region was calculated. The averaged expression level of each gene in two regions connecting a structural covariance edge was considered as the expression level of this gene on this edge, which was used for subsequent analyses.

##### Z-Statistic Maps

As only brain expression data of samples in the left hemisphere were included, we constructed a 7-by-7 matrix (7 × 7 left subcortical regions) and selected 21 non-repeating intrahemispheric edges of the left hemisphere, respectively, for psychotic, disorganized, and total symptom dimensions. We then used *Z*-test to compare the baseline variance of structural covariance for each of the 21 edges between responders and non-responders for each symptom dimension. After that, each edge had a corresponding *Z*-value, and then the *Z*-statistic map of the 21 edges was generated for each symptom dimension. The *Z*-statistic maps were used to represent the variance of baseline subcortical structural covariance related to the variations in treatment response.

##### Partial Least Squares Regression Analysis

We used the partial least squares (PLS) regression, which was previously used in other studies (Morgan et al., 2019; Li et al., 2021), to detect the spatial associations between the baseline *Z*-statistic map and gene expression values of the 21 edges for each symptom dimension. Gene expression data were set as predictor variables, and the *Z*-statistic maps were response variables. The first component in the PLS (PLS1) was the linear combination of gene expression values that were most strongly associated with the Z-statistic maps. A permutation test (1,000 times) was utilized to test the null hypothesis that PLS1 explained no more covariance between the brain-wide expression scores and *Z*-statistic maps than expected by chance. Bootstrapping was used to evaluate each gene’s weight in the PLS1. The ratio of the weight of each regional gene expression to its bootstrap standard error was used to calculate the *Z*-values. After FDR correction (*p* < 0.05), we obtained the gene set that reliably contributed to the PLS1.

#### Step 3: Epigenetic Biomarkers

##### Enrichment Analysis

We performed enrichment analysis from genes in the PLS1 weights |*Z*| > 3 (Morgan et al., 2019) (all FDR < 0.05) by using an online tool Metascape^3^ (Zhou et al., 2019; see details in Supplementary Material).

##### DNA Methylation of Gene of Interest

The baseline peripheral DNAm status of GOI, overlapping between genes in the PLS1 and 108 schizophrenia candidate gene loci previously reported (Schizophrenia Working Group of the Psychiatric Genomics, 2014), was examined in parallel with MRI scanning. Among the participants of this study, 38 controls and 38 patients provided whole blood samples. CpG sites in GOI were selected from the Illumina 450K GeneChip. Briefly, the whole-genome methylation status was then examined in the above 76 samples by using the Illumina 450K GeneChip. This dataset has been previously used by our group (Hu et al., 2020). We used the averaged value of all CpG sites in a gene to represent the DNAm level of this gene. Detailed information about DNA extraction, bisulfite conversion, Illumina 450K GeneChip analysis, the QC controls of the GeneChip assay, and microarray data processing was shown in Supplementary Materials and Methods.

### Associations Between DNA Methylation of Gene of Interest and Treatment Response

We utilized the stepwise multiple linear regression (SMLR) analysis to compute the correlations between patients’ baseline DNAm of GOI and the longitudinal clinical symptom alterations. The independent variables were the DNAm values of GOI, and the dependent variables were the reduction rates (Leucht et al., 2010) of each symptom dimension, i.e., (baseline score − follow-up score)/(baseline score − minimum score in each symptom dimension) × 100%. We used the Kolmogorov–Smirnov test to check the normality distribution of dependent variables and the residuals in the regression model of the SMLR analysis.

### Statistical Analysis

Demographic and clinical behavior data in patients (responders and non-responders at baseline) and healthy volunteers were compared by using a one-way ANOVA test, *t*-test, or chi-squared test.

## Results

### Demographics and Clinical Behaviors

No significant differences were found in the between-group comparisons of the demographic characteristics of 38 healthy volunteers and 38 patients (Table 1). Patients showed a significant clinical improvement in positive, general psychopathology, and total symptoms (all *p* < 0.001; Table 1).

No significant differences were found in between-group comparisons of demographic data among responders, non-responders, and healthy volunteers (all *p* > 0.05, Supplementary Tables 1–3) in the three symptom dimensions.

### Between-Group Comparisons of Baseline Subcortical Structural Covariance for Total Symptoms

**FIGURE 2:**
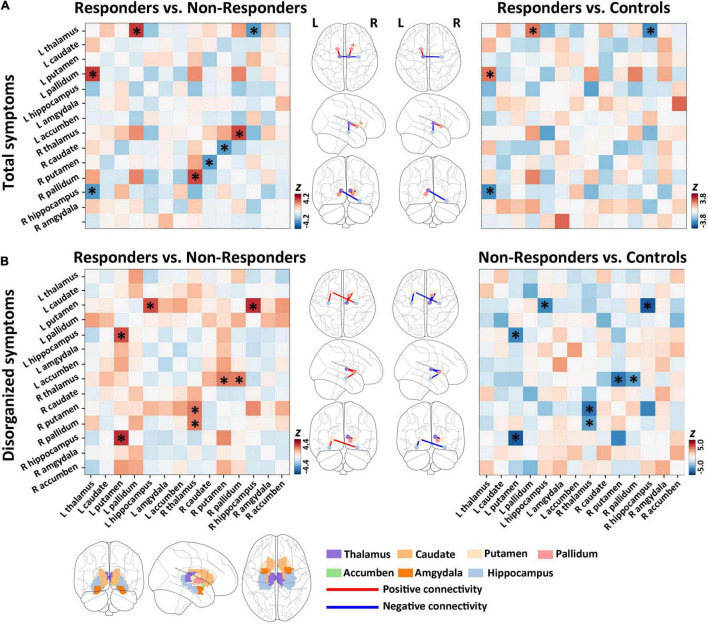
Group differences of baseline subcortical structural covariance for total and disorganized symptoms. **(A)** In the total symptom dimension, we detected significant differences in baseline structural covariance in the hippocampus–thalamus–pallidum–caudate–putamen pathway between responders and non-responders. **(B)** For disorganized symptoms, significant differences in baseline structural covariant connections were found in the putamen–hippocampus–pallidum–thalamus circuit between responders and non-responders.

In comparison with non-responders (*n* = 24), responders (*n* = 14) had significantly higher structural covariance between the right thalamus and the right pallidum (Figure 2A and Supplementary Table 4; *p* = 0.003, *Z* = 3.99, FDR corrected), and between the left thalamus and the left pallidum (Figure 2A and Supplementary Table 4; *p* = 0.002, *Z* = 4.20, FDR corrected), whereas a lower structural covariance was found between the left thalamus and the right hippocampus (Figure 2A and Supplementary Table 4; *p* = 0.021, *Z* = − 3.39, FDR corrected), and between the right putamen and the right caudate (Figure 2A and Supplementary Table 4; *p* = 0.023, *Z* = −3.29, FDR corrected).

Compared with healthy controls, responders had significantly higher structural covariance between the left thalamus and the left pallidum (Figure 2A and Supplementary Table 4; *p* = 0.033, *Z* = 3.19, FDR corrected), but lower structural covariance between the left thalamus and the right hippocampus (Figure 2A and Supplementary Table 4; *p* = 0.014, *Z* = −3.79, FDR corrected). Non-responders showed no significant between-group differences in the structural covariance when compared with healthy controls (all *p* > 0.05 with FDR correction).

### Between-Group Comparisons of Baseline Subcortical Structural Covariance for Disorganized Symptoms

Relative to non-responders (*n* = 11), responders (*n* = 27) had significantly higher structural covariance between the left putamen and the right hippocampus (Figure 2B and Supplementary Table 5; *p* = 0.001, *Z* = 4.407, FDR corrected), between the right thalamus and the right putamen (Figure 2B and Supplementary Table 5; *p* = 0.048, *Z* = 3.116, FDR corrected), between the right thalamus and the right pallidum (Figure 2B and Supplementary Table 5; *p* = 0.048, *Z* = 3.075, FDR corrected), and between the left putamen and the left hippocampus (Figure 2B and Supplementary Table 5; *p* = 0.005, *Z* = 3.883, FDR corrected).

Furthermore, compared with controls, non-responders exhibited significantly lower structural covariance between the left putamen and the right hippocampus (Figure 2B and Supplementary Table 5; *p* < 0.0001, *Z* = − 5.045, FDR corrected), between the right thalamus and the right putamen (Figure 2B and Supplementary Table 5; *p* = 0.0007, *Z* = −4.332, FDR corrected), between the right thalamus and the right pallidum (Figure 2B and Supplementary Table 5; *p* = 0.023, *Z* = −3.226, FDR corrected), and between the left putamen and the left hippocampus (Figure 2B and Supplementary Table 5; *p* = 0.003, *Z* = −3.844, FDR corrected). In addition, responders showed no significant between-group differences in the structural covariance relative to healthy controls (all *p* > 0.05, FDR corrected).

### Between-Group Comparisons of Baseline Subcortical Structural Covariance for Psychotic Symptoms

**FIGURE 3:**
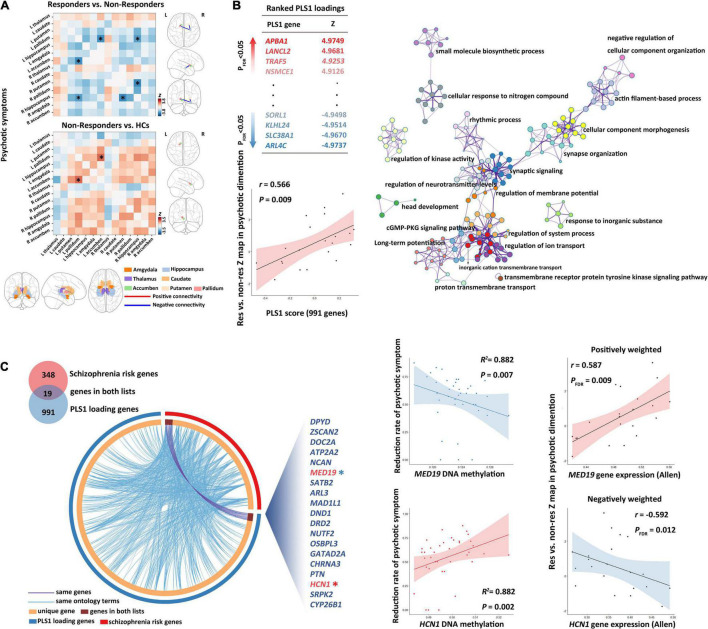
Subcortical connectome, transcriptomic basis, and DNA methylation biomarkers for treatment response of psychotic symptoms. **(A)** Between-group comparisons showed that non-responders had a higher baseline structural covariance in the putamen–hippocampus–pallidum–accumbens pathway compared with responders. **(B)** Partial least squares (PLS) regression analysis demonstrated that expression values of 991 genes in the PLS1 were spatially associated with subcortical connectome variations related to psychotic symptom response. Metascape was then used to align the KEGG pathways and GO biological processes for the PLS1 genes. PLS1 genes were primarily enriched in the neuronal system, ion transport, and cellular process. Each circle node represents an enrichment term. Nodes with the same color belong to the same term (i.e., cluster). The circle node size is equal to the input genes number included in that cluster. **(C)** The DNAm of overlapping genes (shown in the Circos plot) between PLS1 genes and schizophrenia risk genes demonstrated robust correlations with treatment response of psychotic symptoms. GO, gene ontology; KEGG, Kyoto encyclopedia of genes and genomes.

Responders (*n* = 29) had significantly lower structural covariance between the left pallidum and the right hippocampus (Figure 3A and Supplementary Table 6; *p* = 0.035, *Z* = −3.252, corrected by FDR), between the right hippocampus and the right putamen (Figure 3A and Supplementary Table 6; *p* = 0.013, *Z* = −3.807, corrected by FDR), and between the left accumbens and the left pallidum (Figure 3A and Supplementary Table 6; *p* = 0.014, *Z* = −3.616, corrected by FDR) compared with non-responders (*n* = 9).

Moreover, relative to controls, non-responders exhibited significantly higher structural covariance between the left accumbens and the left pallidum (Figure 3A and Supplementary Table 6; *p* = 0.049, *Z* = 3.457, corrected by FDR), whereas responders showed no significant between-group differences in the structural covariance relative to healthy controls (all *p* > 0.05, corrected by FDR).

### Spatial Associations Between Gene Expression and Treatment Response Variances of Psychotic Symptoms

We detected significant PLS components related to the *Z*-statistic map in the psychotic symptom dimension (*r* = 0.566, *p* = 0.0088). We found 991 genes (Figure 3B) with normalized PLS1 weights |*Z*| > 3, including 459 genes with *Z* > 3, and 532 genes with *Z* < −3 (all *p* < 0.001 with FDR correction in Pearson’s correlation analyses for all the 991 genes). The PLS1 explained 30.2% of the variance in the differences in subcortical structural covariance between responders and non-responders.

We did not identify significant PLS components associated with the baseline Z-statistic maps in the total (*p* > 0.05) or disorganized (*p* > 0.05) symptom dimension.

### Enrichment Analysis of First Component in the PLS Genes in the Psychotic Symptom Dimension

The top 20 enriched biological processes and pathways primarily involved 19 biological processes and 1 pathway (cGMP–PKG signaling pathway). Among the 19 biological processes, 7 involved the neuronal system, 3 involved the ion transport processes (with highest Metascape values), 4 involved the cellular processes, and the remaining part primarily involved rhythmic process, metabolic process, and biological regulation. The biological processes in the neuronal system, specifically, included “synaptic signaling,” “head development,” “synapse organization,” “long-term potentiation,” “regulation of neurotransmitter levels,” and “actin filament-based process” (Figure 3B and Supplementary Table 7).

Among the 991 PLS1genes, we detected 19 genes (*ZSCAN2*, *CYP26B1*, *DRD2*, *NCAN*, *DOC2A*, *CHRNA3*, *MED19*, *NUTF2, PTN, DPYD, GATAD2A, OSBPL3, DND1, ARL3, MAD1L1, HCN1, ATP2A2, SATB2*, and *SRPK2*) that overlap with genes in the 108 gene loci (348 genes) (Schizophrenia Working Group of the Psychiatric Genomics, 2014). We defined the 19 overlapping genes as GOI.

In the 19 GOI, 10 genes showed PLS weights *Z* > 3, whereas 9 genes *Z* < −3 (Supplementary Table 8). PLS weights *Z* > 3 (positive associations) represent that genes positively weighted on PLS1 are overexpressed in edges where subcortical structural covariance was increased in responders, whereas PLS weights *Z* < 3 (negatively weighted genes) mean overexpressed in edges where subcortical structural covariance was decreased in responders.

In addition, the schizophrenia risk genes (Schizophrenia Working Group of the Psychiatric Genomics, 2014) were significantly enriched in the 991 PLS1 genes detected in this study (Supplementary Table 9, *p* = 0.0082, chi-square test with Yates’ correction).

### Baseline DNA Methylation of Gene of Interest in Treatment Response of Psychotic Symptoms

In the SMLR analysis, we detected significant correlations between patients’ baseline DNAm of GOI (*HCN1* and *MED19*) and the longitudinal psychotic symptom alterations (*p*-value of the regression model = 7.73 E ^–18^, *F* = 142.671, adjusted *R*^2^ = 0.882, Figure 3C and Supplementary Table 10). Patients’ baseline DNAm of GOI did not show significant associations with the improvement of disorganized or total symptoms.

## Discussion

**FIGURE 4:**
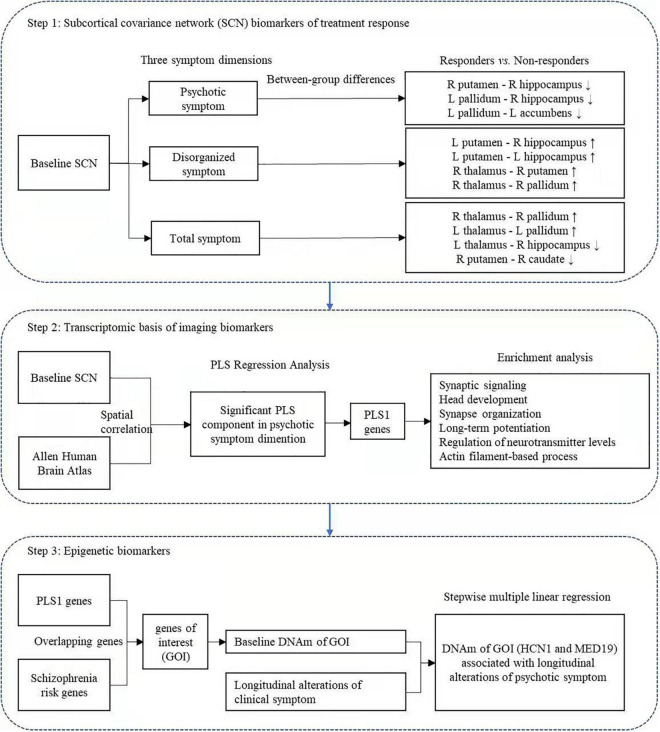
Summary findings corresponding to the three-step multi-omic analysis.

This study is among the first to investigate baseline biomarkers related to treatment outcomes in the early phase of schizophrenia using multi-omic measures linking subcortical covariant network, transcriptomic signatures, and peripheral epigenetic modifications. As hypothesized (Figure 4), the subcortical structural covariance before starting treatment may serve as potential predictors of treatment response in psychotic, disorganized, and total symptoms. Phenotypic variations of subcortical connectome related to psychotic symptom response were spatially associated with the expression of genes primarily enriched in neurobiological processes and the dopaminergic pathway. Moreover, the DNAm of GOI demonstrated significant associations with patients’ improvement of psychotic symptoms.

In the psychotic symptom dimension, non-responders had a higher baseline structural covariance in the putamen–hippocampus–pallidum–accumbens pathway compared with responders, implying that a higher level of structural covariance among these subcortical regions may predict a poor treatment response in this symptom dimension. The above subcortical structures, i.e., the hippocampus, pallidum, accumbens, and putamen, have been proposed to be critical for the reward system (Smith et al., 2009; Mizuno et al., 2016; Legates et al., 2018), which are clearly implicated in the pathophysiology of schizophrenia and pharmacological mechanisms of antipsychotic treatment response (Schultz et al., 1997; Kapur and Mamo, 2003). Our approach demonstrates for the first time that a higher baseline structural covariance in the “subcortical reward circuit” may be associated with a poor response to the psychotic symptoms. It is known that antipsychotic agents bind to dopamine receptors primarily in subcortical structures (Eisenberg et al., 2017). Interestingly, a PET study demonstrated that dopamine synthesis capacity of the striatum explained > 40% of the variance in subsequent changes of psychotic symptoms after treatment, suggesting that dopaminergic dysfunction before starting treatment may underlie future variations in treatment response (Jauhar et al., 2019). We speculate that the predictive effect of the putamen—hippocampus–pallidum–accumbens circuit in this study might be mediated by patients’ altered baseline dopamine levels in these subcortical regions. Further studies in combination with PET and MRI are needed to clarify this speculation.

We also found that subcortical circuit biomarkers are associated with treatment efficacy in disorganized symptoms. Specifically, we found consistently decreased baseline structural covariance in the putamen–hippocampus–pallidum–thalamus circuits in non-responders compared with responders. Many recent studies have reported the involvement of the putamen–hippocampus–pallidum–thalamus loop in the working memory (Kalivas et al., 2001; Karlsgodt et al., 2005). Notably, the disorganized symptoms include linguistic symptoms, such as poverty of speech, poverty of content of speech, tangentiality, and derailment (Andreasen et al., 2005). Evidence shows the associations of disorganized symptoms with verbal working memory (Horan et al., 2008; Torniainen et al., 2012). Our preliminary result implies that consistent reductions of baseline structural covariance in the subcortical verbal working memory circuit may predict a poor response to disorganized symptoms.

Subcortical structural covariance is built on the similarity of macrostructural variations. It reflects structural synchronized maturation and similarities in the local micro-architectonic properties (Alexander-Bloch et al., 2013a,b), which is commonly influenced by many certain factors, such as monosynaptic connection in synapse development (Yee et al., 2018) and gene expression (Raznahan et al., 2011). In this study, we detected significant spatial associations between the variation in subcortical volume covariance related to psychotic symptom response and expression of genes enriched for 19 GO biological processes and 1 KEGG pathway. It is known that treatment response to psychotic symptoms has a significant association with baseline dopamine synthesis capacity of subcortical regions (Jauhar et al., 2019). Interestingly, the identified KEGG pathway, i.e., cGMP–PKG signaling pathway, has been reported to mediate the phosphorylation of DARPP-32 in neostriatal neurons induced by glutamate (Nishi et al., 2005). Significantly, mice lacking DARPP-32, a transduction molecule in dopamine signaling that is selectively enriched in the striatum, exhibit profound deficits in their behavioral responses to antipsychotic medication (Fienberg et al., 1998), which suggests the potential role of the cGMP–PKG pathway in dopaminergic neurotransmission and neuroleptic treatment response. Moreover, the detected GO biological processes were primarily involved in neurobiological processes. Interestingly, the schizophrenia risk genes identified previously (Schizophrenia Working Group of the Psychiatric Genomics, 2014) were significantly enriched in the 991 PLS1 genes detected in this study, implying a common genetic basis emerging for both schizophrenia pathology and response-related subcortical covariant network phenotypes. Consistently, a recent large-scale GWAS-based imaging-genetic study detected evidence of genetic overlap between subcortical brain measures and schizophrenia risk (Smeland et al., 2018).

However, we did not detect spatial correlations in the total or disorganized symptom dimension, suggesting that the phenotypic variations of subcortical connectome related to treatment response of the two symptom dimensions may not be transcriptomically underlaid. Future studies are still needed to validate this speculation.

Importantly, we also detected significant correlations between patients’ baseline DNAm of GOI, i.e., *HCN1* and *MED19*, and the longitudinal psychotic symptom alterations. These associations support a functional role for the peripheral DNAm alterations and suggest the validity of patient classification based on subcortical volumetric covariance network markers. These findings, combined with the above transcriptomic basis of variations in subcortical connectome, imply that treatment response to psychotic symptoms may be different from that of other dimensions, as it may have more robust molecular bases. Noteworthily, *HCN1*, coding for hyperpolarization-activated cyclic nucleotide-gated channels, has been implicated in modulation of dopamine signaling (Rampino et al., 2019). *MED19* is proposed to be involved in cancer growth, and its expression inhibits the spread and growth of cancers (Li et al., 2011; Zhu et al., 2013). This study first reveals the associations between baseline DNAm of these two genes and psychotic symptom improvement. Combining brain connectomes, gene transcripts, and DNAm could provide insight into how macroscale brain connectivity impairments are driven by the microscale architecture.

Our results must be interpreted in light of several limitations. First, the sample size of responders and non-responders in each symptom dimension is relatively small due to the challenging requirement of first-episode drug-naïve patients given risperidone monotherapy. Replication in other datasets with a large sample size is needed. Second, we only included tissue samples in the left hemisphere, as only two of the six donors in the AHBA have brain tissue samples in the right hemisphere. Thus, the association between gene expression and treatment response-related alterations in subcortical structural covariance does not represent the condition of bilateral subcortical structures. Third, the DNAm status was derived from peripheral cells rather than brain tissue samples of the subcortical structures, although DNAm sites were moderately and robustly associated between brain and blood (Braun et al., 2019).

## Conclusion

This study links structural and molecular alterations relevant to therapeutic response in the early phase of schizophrenia. Subcortical structural covariance and peripheral DNAm could be useful predictors of antipsychotic treatment response, and these results are important for future precision medicine. This study also defines a roadmap for future studies investigating multimodal imaging epigenetic biomarkers for treatment response in schizophrenia.

## Data Availability Statement

The datasets presented in this study can be found in online repositories. The names of the repository/repositories and accession number(s) can be found in the article/Supplementary Material.

## Ethics Statement

The studies involving human participants were reviewed and approved by the Medical Ethics Committee of Henan Mental Hospital and Second Xiangya Hospital of Central South University. The patients/participants provided their written informed consent to participate in this study.

## Author Contributions

XZ and ML: study design. XZ, TY, MH, and ZL: data acquisition. CH, XH, JX, LL, ML, XD, and JZ: statistical analysis. XZ, CH, and ML: manuscript writing. All authors have read and agreed to the published version of the manuscript.

## Conflict of Interest

The authors declare that the research was conducted in the absence of any commercial or financial relationships that could be construed as a potential conflict of interest.

## Publisher’s Note

All claims expressed in this article are solely those of the authors and do not necessarily represent those of their affiliated organizations, or those of the publisher, the editors and the reviewers. Any product that may be evaluated in this article, or claim that may be made by its manufacturer, is not guaranteed or endorsed by the publisher.

## Footnotes

1
http://surfer.nmr.mgh.harvard.edu/

2
http://www.brain-map.org

3
https://metascape.org
