# Supplementary material for: Predictive Biomarkers for Antipsychotic Treatment Response in Early Phase of Schizophrenia: Multi-Omic Measures Linking Subcortical Covariant Network, Transcriptomic Signatures, and Peripheral Epigenetics
Source: Front Neurosci. 2022 May 9;16:853186. doi: 10.3389/fnins.2022.853186 (PMC9125083; doi:10.3389/fnins.2022.853186)
Supplement: Supplementary file 1 [file Data_Sheet_1.docx]

**Predictive Biomarkers for Antipsychotic Treatment Response in Early Phase of Schizophrenia: Multi-omic Measures Linking Subcortical Covariant Network, Transcriptomic Signatures and Peripheral Epigenetics**

**Supplementary** **Materials and Methods**

Participants

Any participant with a history of severe physical diseases, neurological disorders, drug abuse or alcohol abuse was excluded from this study. We also exclude participants with abnormal ECG and EEG results, as well as abnormal blood tests including complete blood count test, and comprehensive metabolic panel tests measuring glucose level, cholesterol, electrolyte, kidney function, and liver function.

After the 8-week experiment, patients with satisfactory response were recommended to continue risperidone therapy, while others with unsatisfactory response may need to try second line treatment as recommended by their doctors.

**Antipsychotic Therapy and Clinical Behavior Assessment**

The initial daily dose of risperidone was 1–2 mg. A slow titration protocol was applied: after week 1, dose increases occurred at 1-week intervals until patients improved. For those subjects who did not exhibit significant clinical improvement after week 4, they were allowed to reach a maximum dose of 6 mg/d. All 38 patients received risperidone monotherapy for 8 weeks. Antidepressants and mood stabilizers were not used. The severity of patients’ symptoms was assessed before and after 8 weeks of treatment on the day of MRI scans by using the Positive and Negative Syndrome Scale (PANSS), which includes positive symptoms (PANSS-P, items P1-P7), negative symptoms (PANSS-N, items N1-N7), general psychopathology symptoms (PANSS-G, items G1-G16), and PANSS total symptoms (PANSS-T, all the 30 items) ^1^. The safety of risperidone treatment was evaluated weekly through clinical interviews.

**Treatment response assessment**

Treatment response after 8 weeks of risperidone treatment was assessed according to RSWG.^2^ This group identified the total symptom dimension according to dimensions including psychopathology symptom (i.e., the sum of psychotic items P1, P3 and G9, disorganized items P2 and G5, and negative symptoms items N1, N4 and N6). They defined subjects as responders (this study defined these responders as total symptoms responders) if they had all item scores of the three dimensions ≤3 for at least 6 months. This study applied the remission criteria of RSWG without using the proposed 6 months of the minimum time threshold. Moreover, to explore the treatment response predictors of these three different dimensions aside from the total symptom dimension, we also defined patients as psychotic symptom responders and non-responders, as well as disorganized symptom responders and non-responders, respectively according to the dimensions of psychotic (all item scores of P1, P3 and G9 ≤3), and disorganized (item scores of both P2 and G5 ≤3). Therefore, all schizophrenic subjects at baseline were classified according to their subsequent response. Detailed demographic and clinical characteristics of responders and non-responders at baseline in total, psychotic and disorganized symptom groups are shown in Table S1, S2 and S3.

**T1 Imaging Acquisition**

Data were scanned on a 3.0T Siemens MRI scanner (Verio) at the Magnetic Imaging Centre of the Henan Mental Hospital. A standard 16-channel head coil was used. T1-weighted images were acquired sagittally with a spoiled gradient echo pulse sequence with the following parameters: repetition time (TR) = 1900 ms, echo time (TE) = 2.52 ms, flip angle = 9°, field of view = 250 mm × 250 mm, slice thickness = 1.0 mm, slice gap = 0 mm, and 176 slices.

**Image Analysis**

Subcortical volume estimation was automatically performed with the publicly available FreeSurfer software package by using the segmentation procedure (v5.3.0; <http://surfer.nmr.mgh.harvard.edu/>). Image processing includes motion correction, skull stripping, automated Talairach transformation, and segmentation of subcortical white matter and deep gray matter volumetric structures. In the automated segmentation procedure, FreeSurfer was used to automatically label subcortical tissue classes on the basis of an atlas-based Bayesian segmentation procedure. A subject-independent probabilistic atlas was precalculated from a training set of subjects whose brains were previously labeled. These labels and intensity values were mapped into Talairach space to guarantee voxel-wise correspondence across subjects. A probabilistic atlas was then generated after three types of probabilities were computed for each voxel in Talairach space to encode probabilities of class labels, profiles of intensity values, and function of neighborhood patterns. When the available atlas was created, the segmentation of individual brain MRI could be conducted as follows: Preprocessing steps were composed of affine registration with Talairach space, intensity normalization, skull strip, and a high-dimensional nonlinear volumetric alignment to the Talairach atlas. FreeSurfer computed the probability of a class at each voxel location as the probability that the given class appeared at that location in the training set times the likelihood of obtaining the subject-specific intensity value from that class. An initial segmentation was created by assigning each point to the class where the above probability was greatest. The neighborhood function was then applied to recompute the class probabilities, and the data were resegmented using the new class probabilities. This procedure was repeated if the results did not converge or stabilize. These technical details of these procedure were described in prior publications.^3^ The FreeSurfer analysis pipeline was conducted without any manual intervention.

**Preprocessing of Allen Human Brain Atlas (AHBA) Data**

The expression data were preprocessed according to the 5 major steps: (i) verifying probe-to-gene annotations; (ii) filtering probes not exceeding background noise; (iii) selecting representative probes to index expression for each gene; (iv) assigning and mapping samples to the D–K 68 atlas; (v) normalizing expression values to account for outlying values and inter-participant variances. The code concerning the preprocessing procedure is available at github (https://github.com/BMHLab/AHBAprocessing respectively).

*Characteristics of AHBA Data* The gene expression datasets were extracted from the post-mortem brain tissues of six donors. The donors were a 24-year-old (H0351.2001) and a 39-year-old (H0351.2002) African American male; a 57-year-old (H0351.1009), a 31-year-old (H0351.1012) and a 55-year-old (H0351.1016) European-ancestry male; as well as a 49-year-old Hispanic female (H0351.1015). Further details concerning the gene expression data are also provided at http://www.brain-map.org.

**Enrichment analysis**

Metascape (https://metascape.org) provides automated online meta-analysis to obtain either unique or common enriched pathways in 40 independent knowledge bases. PLS1 gene names was input into the Metascape website, and enrichment terms were filtered to those pathways and biological processes that met a P value < 0.05 with FDR correction. We aligned the Kyoto encyclopedia of genes and genomes (KEGG) pathways and gene ontology (GO) biological processes with the gene list of the PLS1.

**DNA Extraction**

DNA was isolated using QIAamp DNA Blood Mini Kit (Qiagen; Germantown, MD). The A260/A280 ratio measured by utilizing a NanoDrop (Thermo Scientific) was used to assess DNA purity. Agarose gel electrophoresis was used to check DNA quality.

**Bisulfite Conversion and Illumina 450K Genechip Analysis**

Genome-wide DNAm was treated with sodium bisulfite and then quantified respectively by using the EZ DNAm kit (Zymo Research; Irvine, CA) and Infinium® Human Methylation 450K BeadChip (Illumina Inc.; San Diego, CA). According to the recommendations of EZ DNAm Kit manufacturer (Zymo Research; Irvine, CA), 1 μg DNA was utilized for sodium bisulfite-treated assay.

We randomly distributed the two groups to different arrays in order to control the batch effects. DNA methylation level was measured using the Illumina Infinium HD Methylation Assay (Illumina) according to the manufacturer's instructions. We downloaded the manifest files of each array in advance and imaged the BeadChips using an Illumina Scan.

**The QC Controls of Genechip Assay**

The QC controls of all samples from the two groups were tested by using the methylation module of the GenomeStudio software (v1.9). The controls included sample-dependent (bisulfite conversion, stringency, non-polymorphic and negative controls), as well as sample-independent controls（staining, extension, target removal and hybridization controls). We qualified the above controls according to the Illumina’s instruction.

**Illumina 450K Microarray Data Processing**

Raw data of Illumina array was processed by using the methylation module of the GenomeStudio (v1.9) based on default parameters, which outputted signal intensities and detection P-values of all probes. We then filtered the samples which had more than 10% CpG sites with detected P > 0.05 and probes which had more than 25% samples with detected P > 0.05. Raw data were normalized by utilizing BMIQ R packages and Lumi including probe type bias and color-bias adjustment. We used Lumi to convert the individual sites for each sample into methylation levels, i.e., β-values, which ranged from 0 to 1 and were computed as the ratio of the DNA methylation signal intensity to the sum of both unmethylated and methylated signals.

**Supplementary Results**

**TABLE S1** Demographic and clinical characteristics among responders and non-responders in the total symptom dimension and healthy controls.

| **Variables** | **Responders**  **(*n* = 14)** | **Non-responders**  **(*n* = 24)** | **Healthy Controls**  **(*n* = 38)** | ***t*/*F*/χ^2^** **^a^** | ***P*** |
| --- | --- | --- | --- | --- | --- |
| Age (years, mean ± SD) | 27.36 ± 4.24 | 23.63 ± 4.89 | 24.76 ± 4.56 | *F*_(2,73)_ = 2.92 | 0.06 |
| Education (months, mean ± SD) | 11.14 ± 3.11 | 9.96 ± 2.68 | 11.05 ± 2.91 | *F*_(2,73)_ = 1.25 | 0.29 |
| Duration of psychosis (months, mean ± SD) | 7.85 ± 2.48 | 8.46 ± 2.70 | NA | *t*_(36)_ = −0.68 | 0.50 |
| Handedness  (R/L) | 14/0 | 24/0 | 38/0 | NA | NA |
| Gender  (M/F) | 10/4 | 15/9 | 25/13 | χ^2^_(2)_ = 0.31 | 0.86 |
| PANSS-T (mean ± SD) | 92.29 ± 13.83 | 93.25 ± 9.22 | NA | *t*_(36)_ = −0.26 | 0.80 |
| PANSS-P (mean ± SD) | 26.36 ± 3.82 | 25.75 ± 3.54 | NA | *t*_(36)_ = 0.50 | 0.62 |
| PANSS-N (mean ± SD) | 17.93 ± 5.74 | 18.54 ± 4.78 | NA | *t*_(36)_ = −0.35 | 0.73 |
| PANSS-G (mean ± SD) | 48 ± 6.88 | 48.96 ± 6.43 | NA | *t*_(36)_ = −0.43 | 0.67 |

Abbreviations: L = left; R = right; M = male; F = female; SD = standard deviation; PANSS = Positive and Negative Syndrome Scale; PANSS-T = PANSS total symptoms; PANSS-P = PANSS positive symptoms; PANSS-N = PANSS negative symptoms; PANSS-G, PANSS general psychopathology symptoms; NA = not applicable.

^a^ *t*(df), Between-group *t* statistic and degrees of freedom. χ^2^_(df)_, Between-group chi-square statistic and degrees of freedom. *F*_(dfn, dfd)_, one-way ANOVA test and degrees of freedom numerator and degrees of freedom denominator.

**TABLE S2** Demographic and clinical characteristics among responders and non-responders in the psychotic symptom dimension and healthy controls.

| **Variables** | **Responders**  **(*n* = 29)** | **Non-responders**  **(*n* = 9)** | **HC**  **(*n* = 38)** | ***t*/*F*/χ^2^** **^a^** | ***P*** |
| --- | --- | --- | --- | --- | --- |
| Age (years, mean ± SD) | 25.38 ± 4.63 | 23.78 ± 6.02 | 24.76 ± 4.56 | *F*_(2,73)_ = 0.41 | 0.66 |
| Education (months, mean ± SD) | 10.66 ± 2.97 | 9.56 ± 2.46 | 11.05 ± 2.91 | *F*_(2,73)_ = 0.99 | 0.38 |
| Duration of psychosis (months, mean ± SD) | 8.17 ± 2.58 | 8.45 ± 2.83 | NA | *t*_(36)_ = −1.42 | 0.16 |
| Handedness  (R/L) | 29/0 | 9/0 | 38/0 | NA | NA |
| F/M | 9/20 | 4/5 | 13/25 | *χ^2^*_(2)_ = 0.55 | 0.76 |
| PANSS-T | 93.83 ± 11.65 | 89.89 ± 8.24 | NA | *t*_(36)_ = 0.94 | 0.35 |
| PANSS-P | 25.86 ± 3.41 | 26.33 ± 4.39 | NA | *t*_(36)_ = −0.34 | 0.74 |
| PANSS-N | 18.86 ± 5.21 | 16.56 ± 4.50 | NA | *t*_(36)_ = 1.19 | 0.24 |
| PANSS-G | 49.10 ± 6.21 | 47 ± 7.62 | NA | *t*_(36)_ = 0.84 | 0.41 |

Abbreviations: L = left; R = right; M = male; F = female; SD = standard deviation; PANSS = Positive and Negative Syndrome Scale; PANSS-T = PANSS total symptoms; PANSS-P = PANSS positive symptoms; PANSS-N = PANSS negative symptoms; PANSS-G, PANSS general psychopathology symptoms; NA = not applicable.

^a^ *t*_(df)_, Between-group *t* statistic and degrees of freedom. χ^2^_(df)_, Between-group chi-square statistic and degrees of freedom. *F*_(dfn, dfd)_, one-way ANOVA test and degrees of freedom numerator and degrees of freedom denominator.

**TABLE S3** Demographic and clinical characteristics among responders and non-responders in the disorganized symptom dimension and healthy controls.

| **Variables** | **Responders**  **(*n* = 27)** | **Non-responders**  **(*n* = 11)** | **HC**  **(*n* = 38)** | ***t/F/χ^2^* ^a^** | ***P*** |
| --- | --- | --- | --- | --- | --- |
| Age (years) | 25.15 ± 5.07 | 24.64 ± 4.86 | 24.76 ± 4.56 | *F*_(2,73)_ = 0.07 | 0.93 |
| Education (months) | 10.33 ± 2.88 | 10.55 ± 2.95 | 11.05 ± 2.91 | *F*_(2,73)_ = 0.51 | 0.60 |
| Duration of psychosis (months) | 8.33 ± 2.68 | 8 ± 2.53 | NA | *t*_(36)_ = 0.35 | 0.73 |
| Handedness  (R/L) | 27/0 | 11/0 | 38/0 | NA | NA |
| Gender  (M/F) | 19/8 | 6/5 | 25/13 | *χ^2^*_(2)_ = 0.87 | 0.65 |
| PANSS-T | 91.81 ± 11.54 | 95.54 ± 9.35 | NA | *t*_(36)_ = −0.95 | 0.35 |
| PANSS-P | 25.70 ± 3.78 | 26.64 ± 3.20 | NA | *t*_(36)_ = −0.72 | 0.48 |
| PANSS-N | 18.11 ± 5.15 | 18.82 ± 5.15 | NA | *t*_(36)_ = −0.38 | 0.71 |
| PANSS-G | 48 ± 7.06 | 50.09 ± 4.95 | NA | *t*_(36)_ = −0.89 | 0.38 |

Abbreviations: L = left; R = right; M = male; F = female; SD = standard deviation; PANSS = Positive and Negative Syndrome Scale; PANSS-T = PANSS total symptoms; PANSS-P = PANSS positive symptoms; PANSS-N = PANSS negative symptoms; PANSS-G, PANSS general psychopathology symptoms; NA = not applicable.

^a^ *t*_(df)_, Between-group *t* statistic and degrees of freedom. χ^2^_(df)_, Between-group chi-square statistic and degrees of freedom. *F*_(dfn, dfd)_, one-way ANOVA test and degrees of freedom numerator and degrees of freedom denominator.

**TABLE S4** Between**-**group differences of subcortical structural covariance in the total symptom dimension.

| **Edges** | **Responders vs. Non-responders** | **Responders vs. Controls** | **Non-responders**  **vs. Controls** |
| --- | --- | --- | --- |
| R thalamus - R pallidum | ↑ | -- | -- |
| L thalamus - L pallidum | ↑ | ↑ | -- |
| L thalamus - R hippocampus | ↓ | ↓ | -- |
| R putamen - R caudate | ↓ | -- | -- |

Note: L = left; R = right.

“↑” represents significantly increased structural covariance connectivity. “↓” represents significantly reduced structural covariance connectivity.

**TABLE S5** Between**-**group differences of subcortical structural covariance in the disorganized symptom dimension.

| **Edges** | **Responders vs. Non-responders** | **Responders vs. Controls** | **Non-responders**  **vs. Controls** |
| --- | --- | --- | --- |
| L putamen - R hippocampus | ↑ | -- | ↓ |
| L putamen - L hippocampus | ↑ | -- | ↓ |
| R thalamus - R putamen | ↑ | -- | ↓ |
| R thalamus - R pallidum | ↑ | -- | ↓ |

Note: L = left; R = right.

“↑” represents significantly increased structural covariance connectivity. “↓” represents significantly reduced structural covariance connectivity.

**TABLE S6** Between**-**group differences of subcortical structural covariance in the psychotic symptom dimension.

| **Edges** | **Responders vs. Non-responders** | **Responders vs. Controls** | **Non-responders**  **vs. Controls** |
| --- | --- | --- | --- |
| R putamen - R hippocampus | ↓ | -- | -- |
| L pallidum - R hippocampus | ↓ | -- | -- |
| L pallidum - L accumbens | ↓ | -- | ↑ |

Note: L = left; R = right.

“↑” represents significantly increased structural covariance connectivity. “↓” represents significantly reduced structural covariance connectivity.

**TABLE S7**  The TOP 20 enriched biological processes and pathways of PLS1 genes in the psychotic symptom dimension (*P* < 0.05 with FDR correction).

| **Terms** | **Category（GO/KEGG）** | **Description** | **system or parent term** | **Count** | **%** | **Log10(*P*)** | **Log10(*q*)** |
| --- | --- | --- | --- | --- | --- | --- | --- |
| GO:0098662 | GO Biological Processes | inorganic cation transmembrane transport | ion transport | 70 | 7.07 | -13.00 | -8.79 |
| GO:0099536 | GO Biological Processes | synaptic signaling | neuronal system | 65 | 6.57 | -11.85 | -7.93 |
| GO:0060322 | GO Biological Processes | head development | neuronal system | 63 | 6.36 | -9.89 | -6.45 |
| GO:0043269 | GO Biological Processes | regulation of ion transport | ion transport | 57 | 5.76 | -9.58 | -6.21 |
| GO:0042391 | GO Biological Processes | regulation of membrane potential | Biological regulation | 40 | 4.04 | -8.29 | -5.03 |
| GO:0032989 | GO Biological Processes | cellular component morphogenesis | anatomical structure development | 55 | 5.56 | -7.68 | -4.46 |
| GO:0007169 | GO Biological Processes | transmembrane receptor protein tyrosine kinase signaling pathway | signal transduction | 49 | 4.95 | -7.58 | -4.45 |
| GO:0051129 | GO Biological Processes | negative regulation of cellular component organization | cellular process | 51 | 5.15 | -7.14 | -4.10 |
| GO:1901699 | GO Biological Processes | cellular response to nitrogen compound | cellular process | 51 | 5.15 | -7.10 | -4.09 |
| GO:0050808 | GO Biological Processes | Synapse organization | neuronal system | 37 | 3.74 | -7.06 | -4.08 |
| GO:0043549 | GO Biological Processes | regulation of kinase activity | cellular process | 54 | 5.45 | -7.02 | -4.08 |
| GO:0030029 | GO Biological Processes | actin filament-based process | neuronal system | 55 | 5.56 | -6.66 | -3.80 |
| hsa04720 | KEGG Pathway | Long-term potentiation | neuronal system | 13 | 1.31 | -6.49 | -3.67 |
| ko04022 | KEGG Pathway | cGMP-PKG signaling pathway | - | 20 | 2.02 | -6.37 | -3.60 |
| GO:0044057 | GO Biological Processes | regulation of system process | cellular process | 43 | 4.34 | -6.15 | -3.45 |
| GO:0010035 | GO Biological Processes | response to inorganic substance | neuronal system | 42 | 4.24 | -5.89 | -3.28 |
| GO:0048511 | GO Biological Processes | rhythmic process | rhythmic process | 27 | 2.73 | -5.64 | -3.11 |
| GO:0001505 | GO Biological Processes | regulation of neurotransmitter levels | neuronal system | 22 | 2.22 | -5.62 | -3.10 |
| GO:0044283 | GO Biological Processes | small molecule biosynthetic process | metabolic process | 40 | 4.04 | -5.48 | -2.98 |
| GO:1902600 | GO Biological Processes | proton transmembrane transport | ion transport | 18 | 1.82 | -5.37 | -2.89 |

Abbreviations: KEGG, Kyoto encyclopedia of genes and genomes. GO, gene ontology.

**TABLE S8** The PLS1 weights of 19 overlapping genes.

| **10 genes with *Z* > 3** | **PLS1 weights (*Z*)** | **9 genes with *Z* < -3** | **PLS1 weights (*Z*)** |
| --- | --- | --- | --- |
| *ZSCAN2* | 4.133 | *GATAD2A* | -4.878 |
| *CYP26B1* | 4.085 | *OSBPL3* | -4.33 |
| *DRD2* | 3.884 | *DND1* | -4.297 |
| *NCAN* | 3.767 | *ARL3* | -3.825 |
| *DOC2A* | 3.614 | *MAD1L1* | -3.601 |
| *CHRNA3* | 3.601 | *HCN1* | -3.394 |
| *MED19* | 3.538 | *ATP2A2* | -3.301 |
| *NUTF2* | 3.472 | *SATB2* | -3.041 |
| *PTN* | 3.338 | *SRPK2* | -3.001 |
| *DPYD* | 3.106 |  |  |

PLS1 = the first component in the PLS (partial least squares) regression analysis.

**TABLE S9** Enrichment analysis of schizophrenia risk genes in the 991 PLS1 genes (*P* = 0.0082, *chi-square* test with *Yates'* correction).

|  | **Outcome 1** | **Outcome 2** | **Total** | ***X^2^*** | ***P* with Yates' correction** |
| --- | --- | --- | --- | --- | --- |
| Group 1 | 991^a^ | 9036 | 10027^b^ | 6.995 | *P* = 0.0082 |
| Group 2 | 19^c^ | 329 | 348^d^ |  |  |
| Total | 1010 | 9365 | 10375 |  |  |

^a^ PLS1 gene number; ^b^ background genes, i.e., 10027 AHBA genes after 5 steps of preprocessing;

^c^ overlapping number of PLS1 genes with schizophrenia risk genes ^4^;^d^ schizophrenia risk gene number ^4^.

**TABLE S10** The detailed results in the SMLR analysis.

| **Independent Variables** | **normalized *β*** | ***t*** | ***P*** |
| --- | --- | --- | --- |
| Methylation value of *HCN1* | 7.052 | 3.311 | 0.002 |
| Methylation value of *MED19* | -6.126 | -2.876 | 0.007 |
| Normality distribution of residual in the regression model |  |  | *P* > 0.05^a^ |

Abbreviations: SMLR = stepwise multiple linear regression.

^a^  We used the Kolmogorov-Smirnov test to check the normality distribution of the residuals in the regression model of the SMLR analysis.

**DATA AVAILABILITY STATEMENT**

The datasets presented in this study can be found in online repositories. The names of the repository/repositories and accession number(s) can be found at: H6WORLD, 13498. The website is https://www.h6world.cn/dataSpace/detail/13498. Further inquiries can be directed to Maolin Hu, humaolin@whu.edu.cn.

**References:**

1. Kay SR, Fiszbein A, Opler LA. The positive and negative syndrome scale (PANSS) for schizophrenia. *Schizophr bull* 1987;13:261-76.

2. Andreasen NC, Carpenter WT, Jr., Kane JM, Lasser RA, Marder SR, Weinberger DR. Remission in schizophrenia: proposed criteria and rationale for consensus. *Am J Psychiatry* 2005;162:441-9.

3. Fischl B, Salat DH, Busa E, et al. Whole brain segmentation: automated labeling of neuroanatomical structures in the human brain. *Neuron* 2002;33:341-55.

4. Schizophrenia Working Group of the Psychiatric Genomics C. Biological insights from 108 schizophrenia-associated genetic loci. *Nature* 2014;511:421-27.
